# Supplementary material for: Green Aromatic Epoxidation with an Iron Porphyrin Catalyst for One-Pot Functionalization of Renewable Xylene, Quinoline, and Acridine
Source: Molecules. 2023 May 7;28(9):3940. doi: 10.3390/molecules28093940 (PMC10180454; doi:10.3390/molecules28093940)
Supplement: Supplementary file 1 [file molecules-28-03940-s001.zip › molecules-2316416-supplementary.pdf]

molecules

MDPI

Supplementary Material

Green Aromatic Epoxidation with an Iron Porphyrin Catalyst for One-Pot Functionalization of Renewable Xylene, Quinoline, and Acridine

Gabriela A. Corrêa, Susana L. H. Rebelo * and Baltazar de Castro

LAQV/REQUIMTE, Departamento de Química e Bioquímica, Faculdade de Ciências, Universidade do Porto, Rua do Campo Alegre s/n, 4169-007 Porto, Portugal
* Correspondence: susana.rebelo@fc.up.pt

Table of contents

1. Additional characterization of *o*-Xylene products 2
   1.1. Compound 1a 2
   1.2 Compound 1b 2
2. Additional characterization of quinoline products 5
   2.1. Compounds 2a 5
   2.2. Compounds 2b and 2c 7
3. NMR spectra of a quinoline total reaction mixture 10
4. Additional characterization of acridine products 12
   4.1. Compound 3a and 3b 12
5. Catalyst stability studies 13

1. Additional characterization of *o*-Xylene products

1.1. Compound **1a**

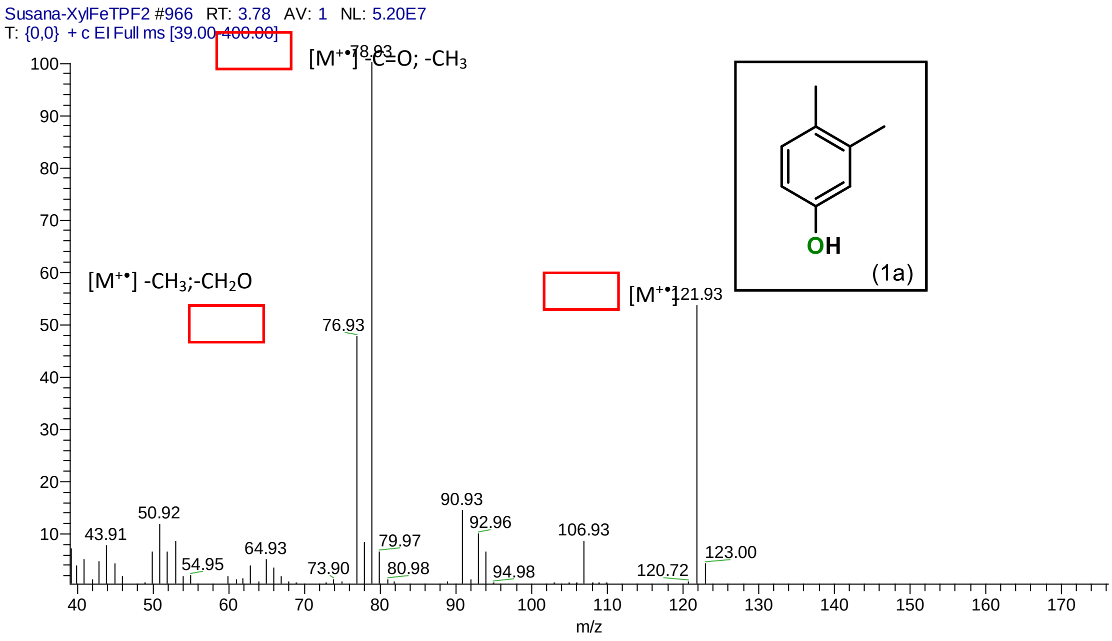

Figure S1. MS spectrum (EI) of product **1a** obtained from GC-MS analysis of *o*-Xylene oxidation reaction.

1.2 Compound 1b

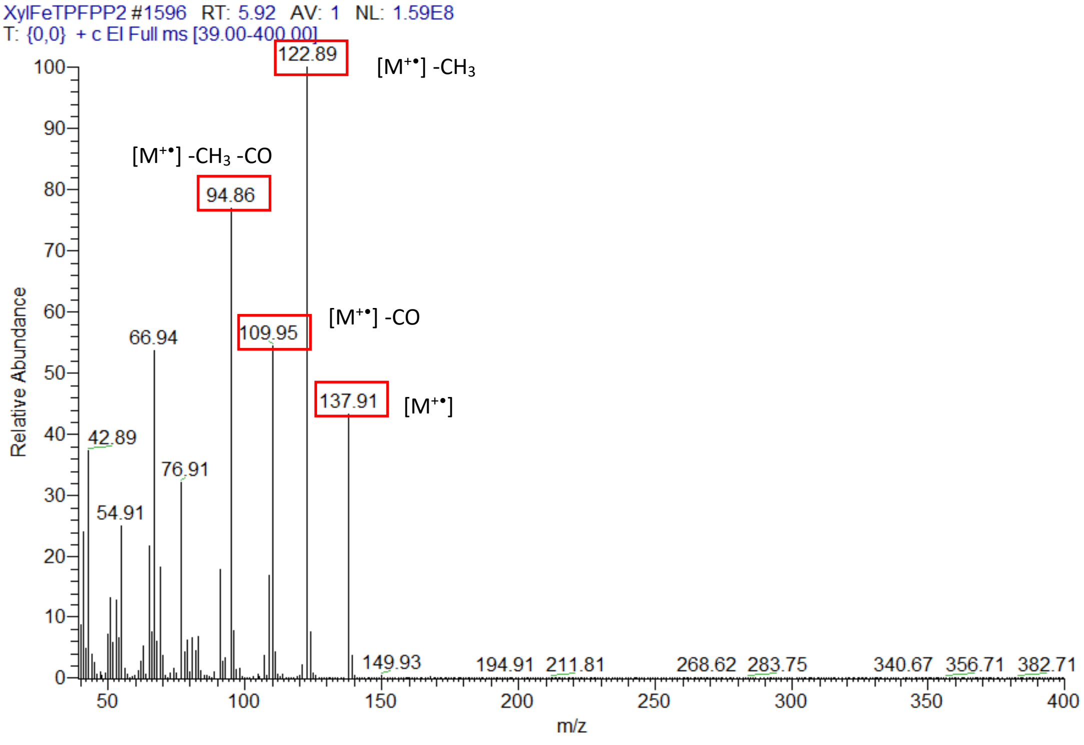

Figure S2. MS spectrum (EI) of product **1b** obtained from GC-MS analysis of *o*-Xylene oxidation reaction.

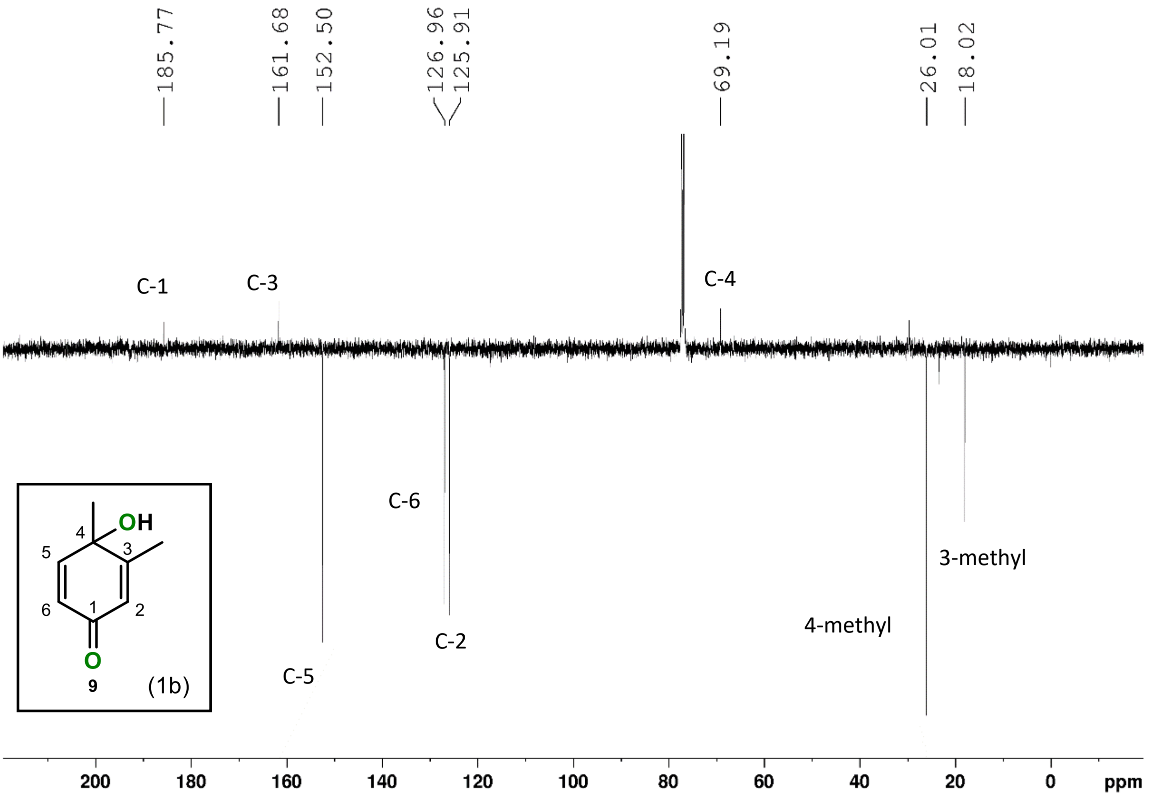

Figure S3. APT spectrum for product **1b** in $CDCl_3$.

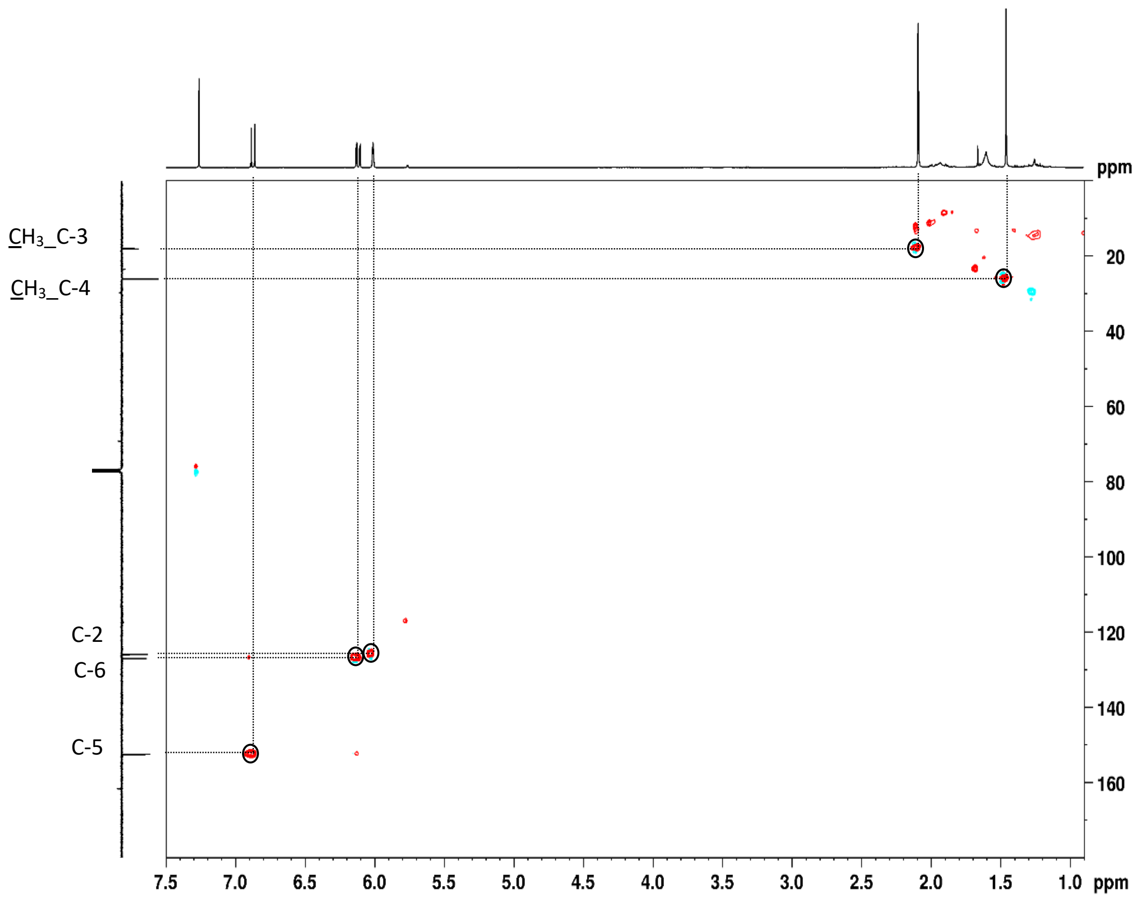

Figure S4. HSQC spectrum for product **1b** in $CDCl_3$.

2. Additional characterization of quinoline products

2.1. Compounds 2a

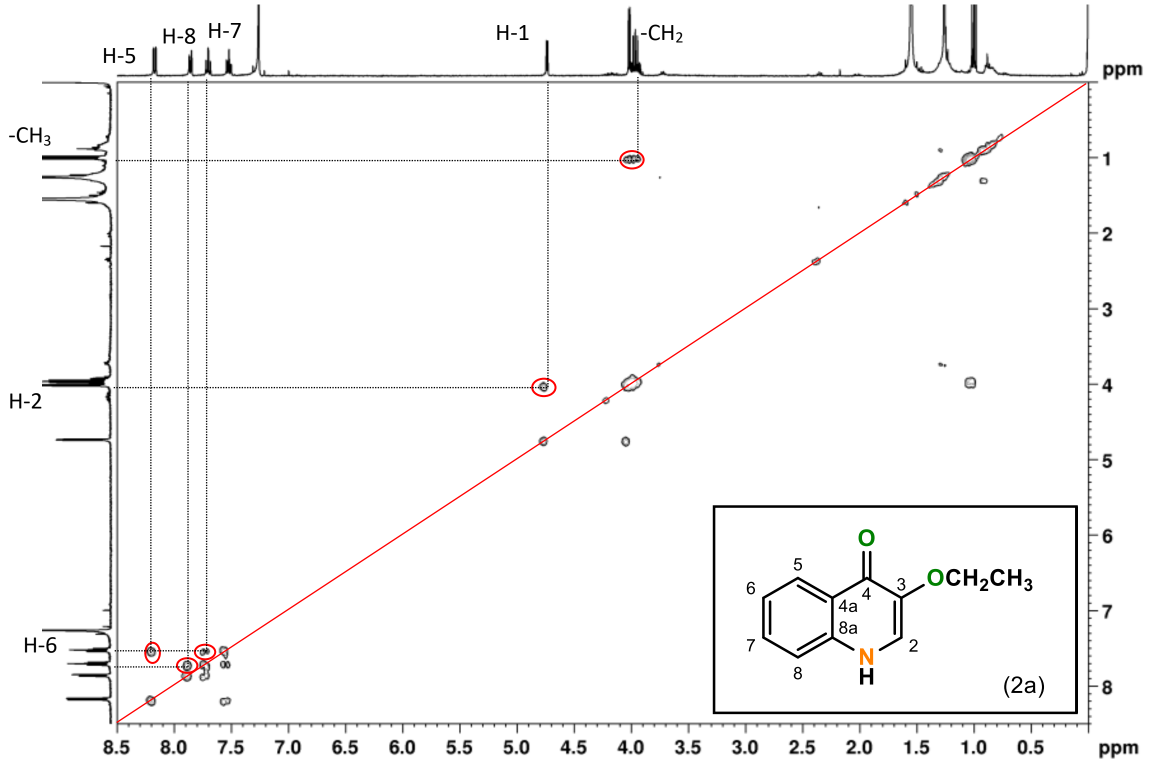

Figure S5. COSY spectrum from product **2a** in $CDCl_3$.

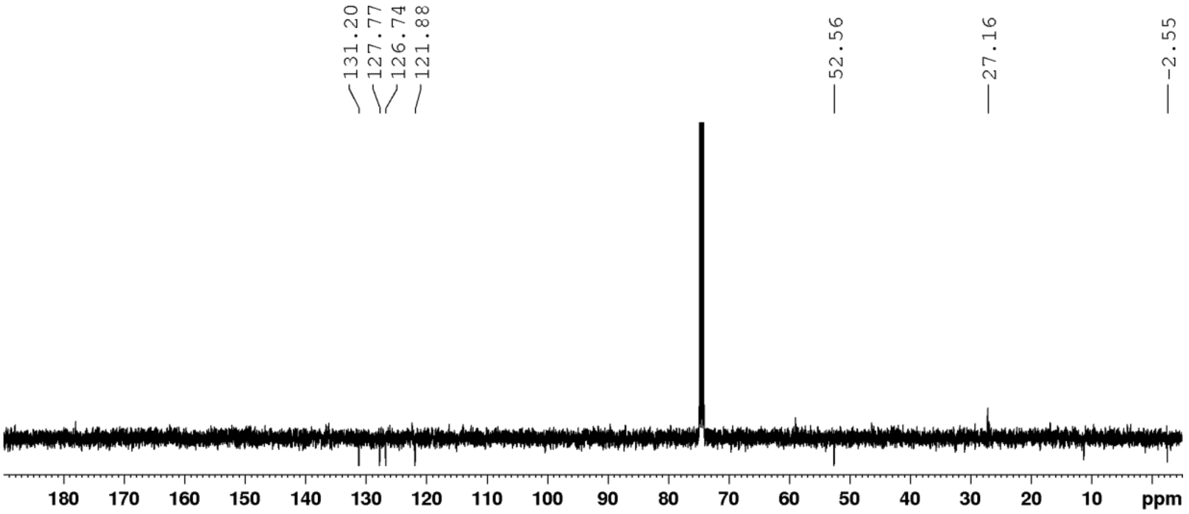

Figure S6. APT spectrum from product **2a** in $CDCl_3$. Mesomerism present in the aza-ring justifies the low intensity of related signals relatively to the signals of the phenyl ring.

2.2. Compounds **2b** and **2c**

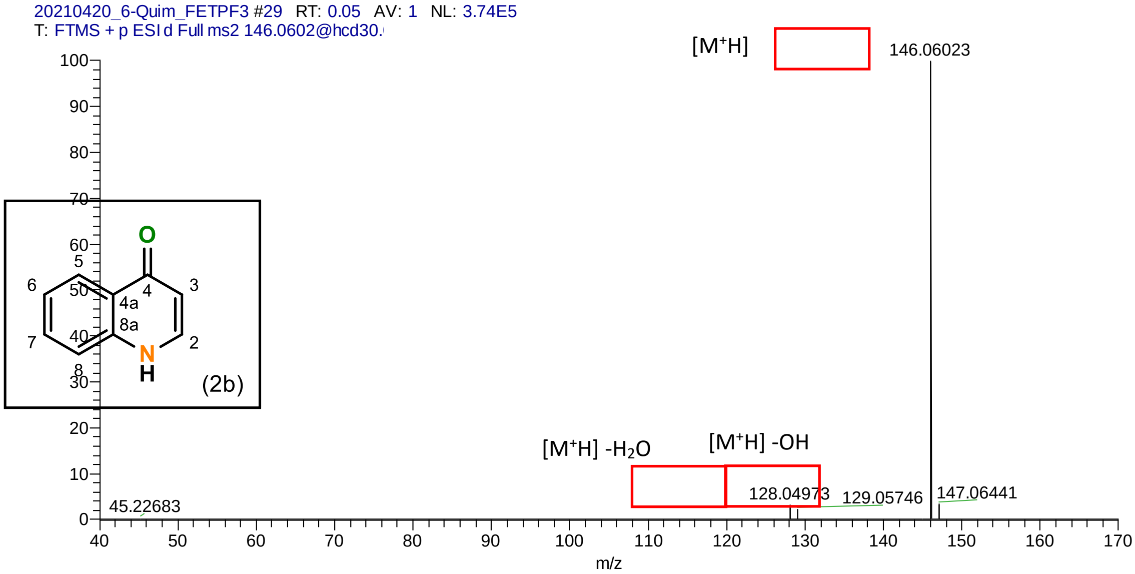

Figure S7. HR-MS^2 (ESI) spectrum from molecular ion corresponding to product **2b** at *m/z* 146.

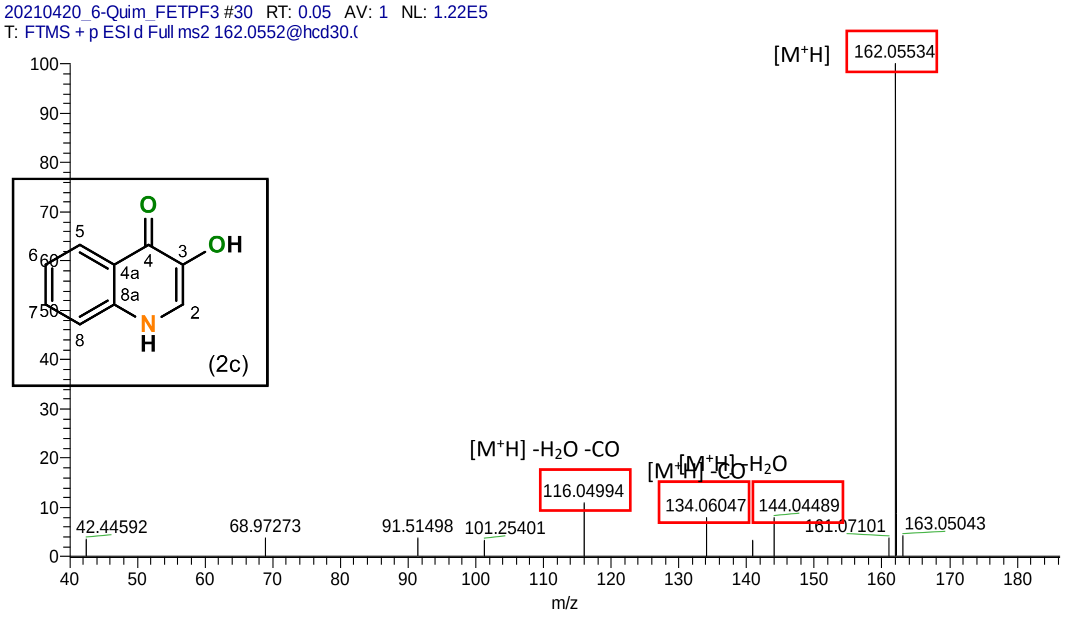

Figure S8. HR-MS^2 (ESI) spectrum from molecular ion corresponding to product **2c** at *m/z* 162.

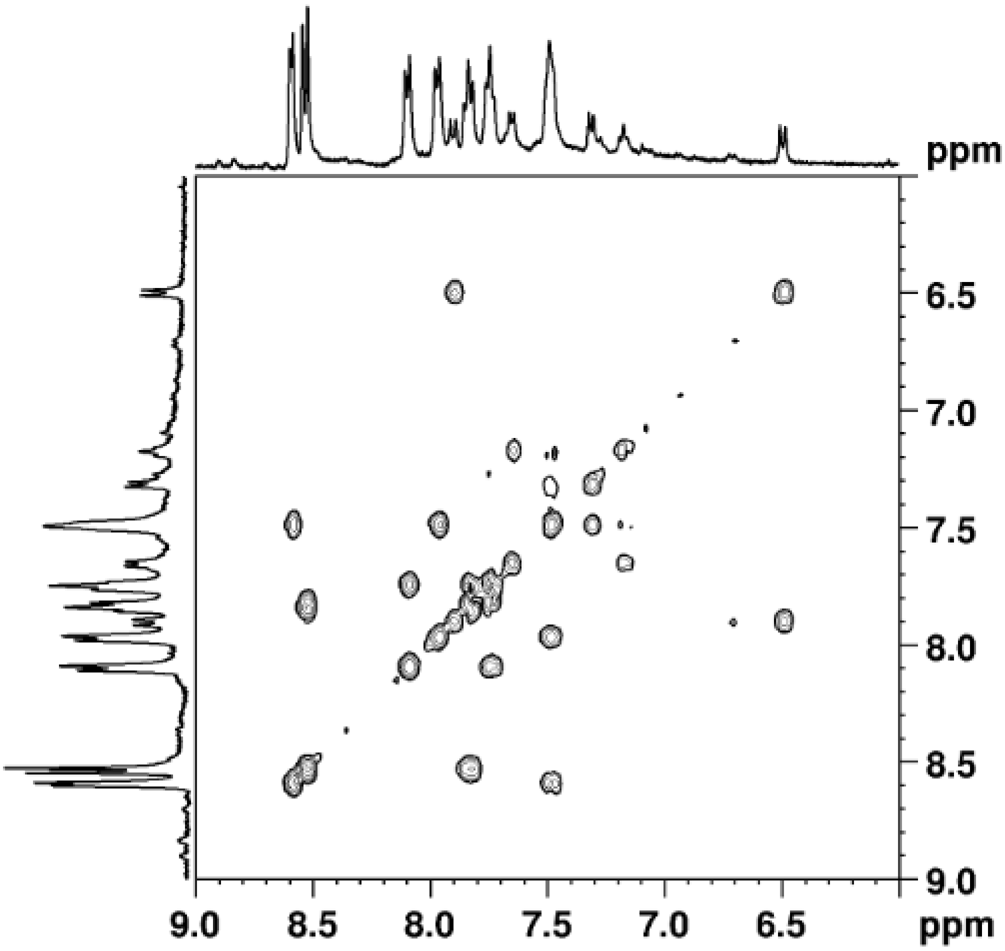

Figure S9. Amplified COSY spectrum in DMSO-d6 of a TLC fraction from a quinoline oxidation reaction under Path II. Compounds **2b**, **2b*** and **2c*** were identified.

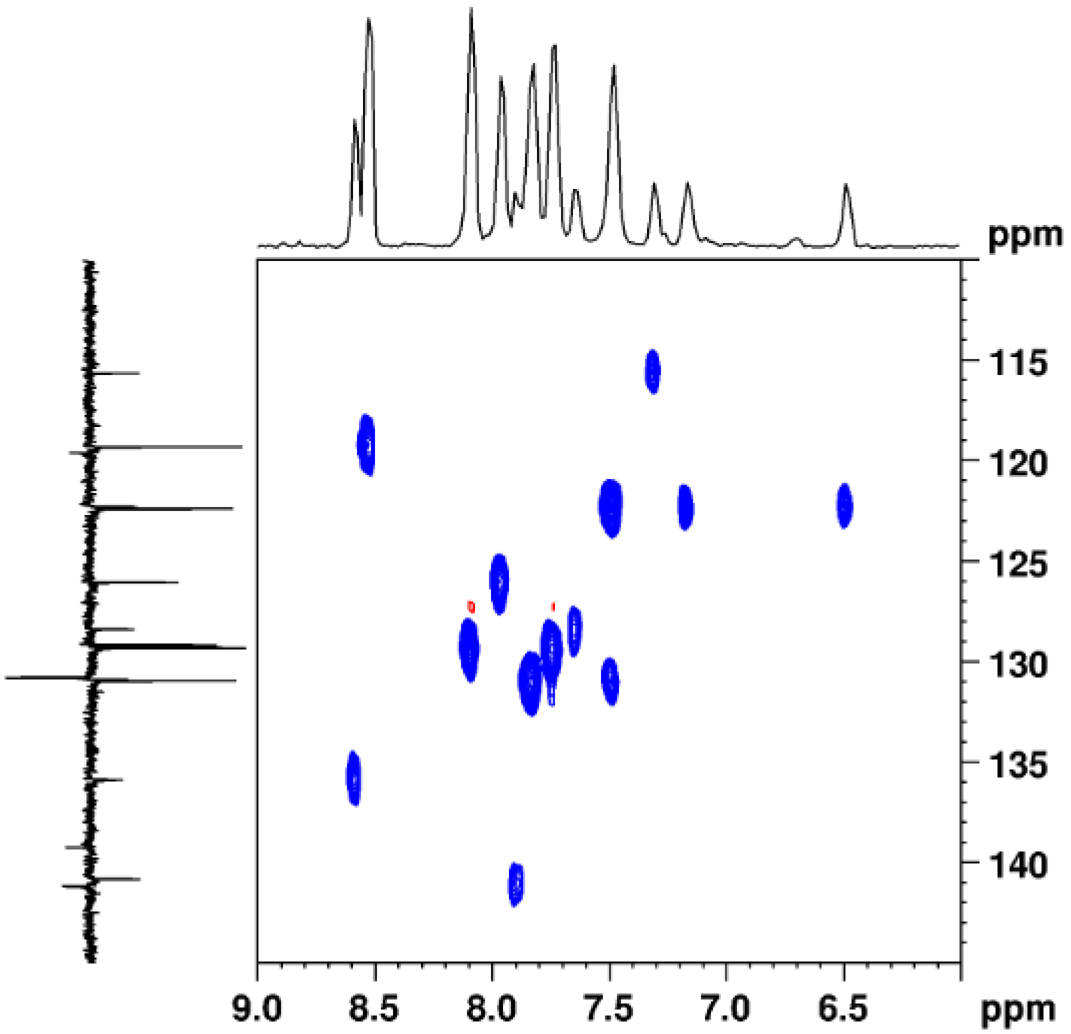

Figure S10. Amplified HSQC spectrum DMSO-d6 of a TLC fraction from a quinoline oxidation reaction under Path II. Compounds **2b**, **2b*** and **2c*** were identified.

3. NMR spectra of a quinoline total reaction mixture

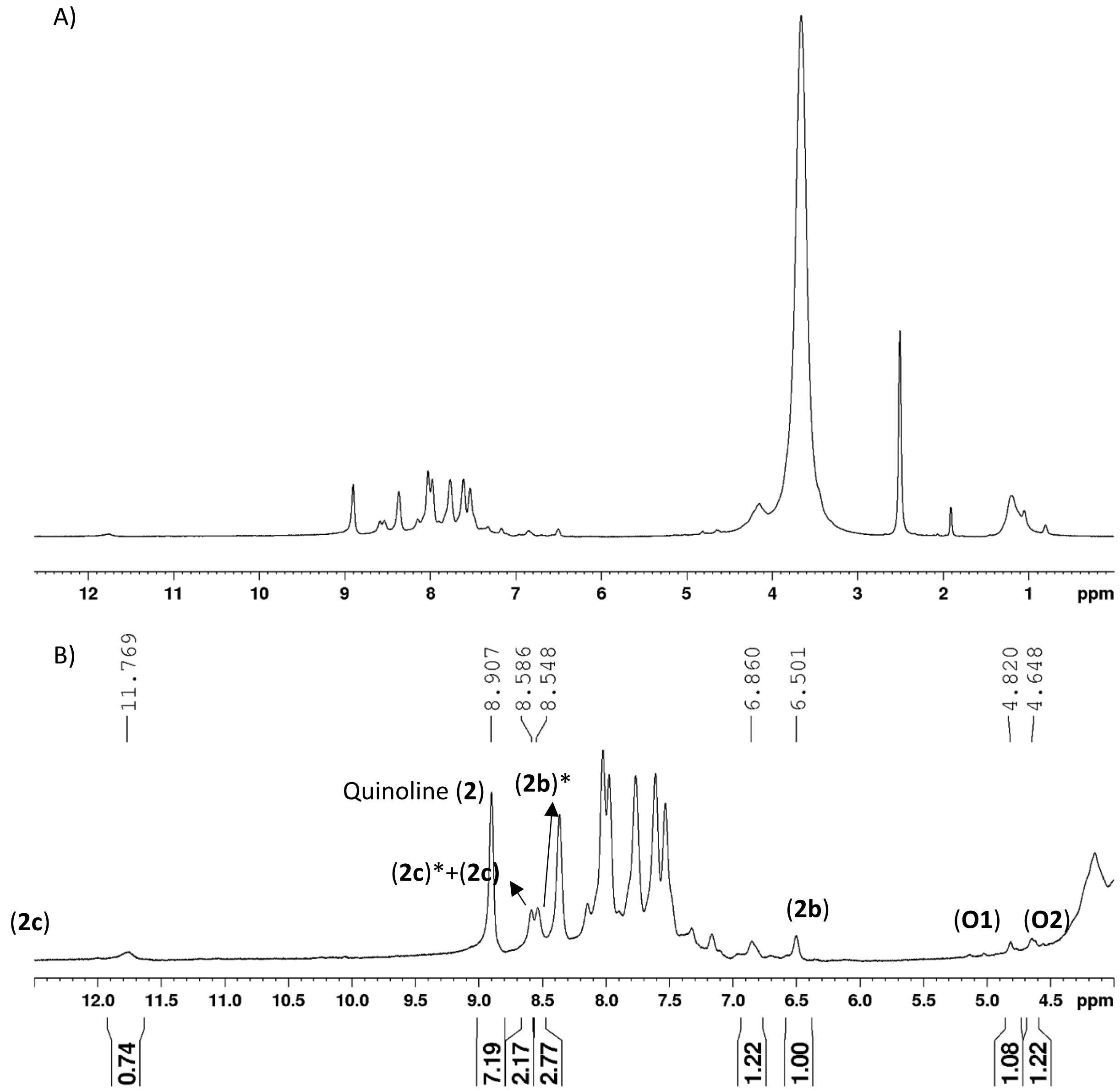

Figure S11. A) Full and B) amplified ^{1}H spectrum of quinoline total reaction mixture in DMSO-d6. The peaks used to quantify the individual products in the reaction mixture are marked: Quinoline (**2**) at δ **8.90** ppm (H-2); compound **2b** at δ **6.50** ppm (H-3, doublet with *J*=9.4 Hz typical of H-*cis* from double bonds, coupling with peak at δ 7.91 ppm); Compound **2b*** at δ **8.55** ppm (H-8); Compound **2c** at δ **11.7** ppm (OH); Compound **2c*** at δ **8.60** ppm (H-2, doublet with *J*=4.8 Hz coupling with OH, minus the area of compound **2c**); Others (**O**) include δ 6.85 ppm ascribed to an intermediate product and δ 4.82 ppm (coupling with peak at δ 4.19 ppm, similarly to compound **2a**);.

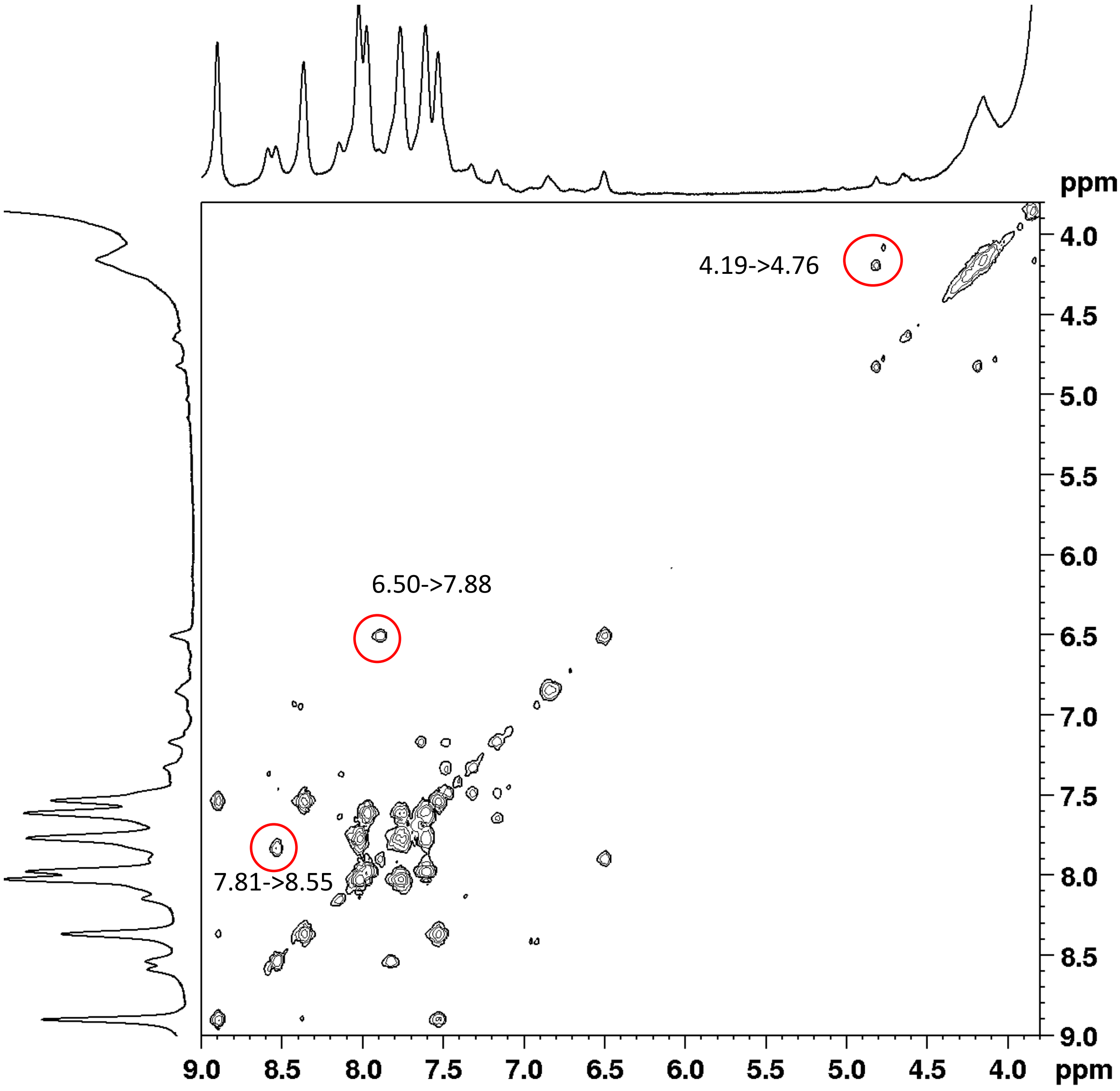

Figure S12. Amplified COSY spectrum in DMSO-d6 of reaction mixture from a quinoline oxidation reaction under Path II.

4. Additional characterization of acridine products

4.1. Compound **3a** and **3b**

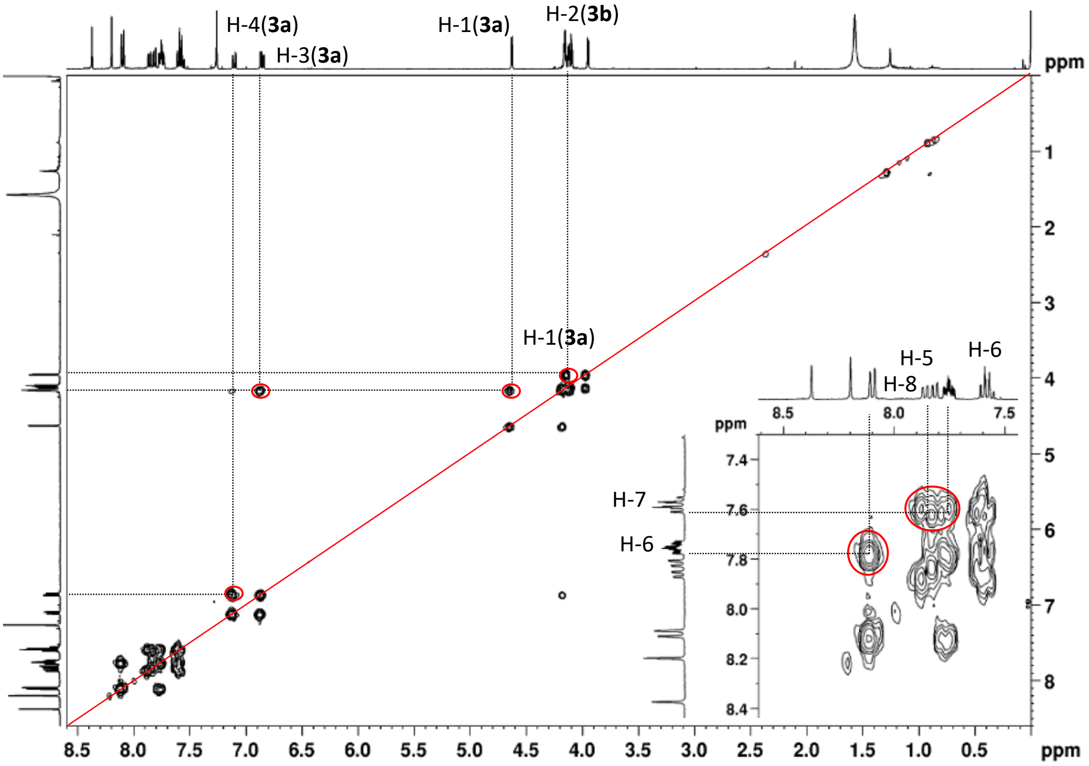

Figure S13. COSY spectrum from a fraction containing a mixture of products **3a** and **3b** in $CDCl_3$.

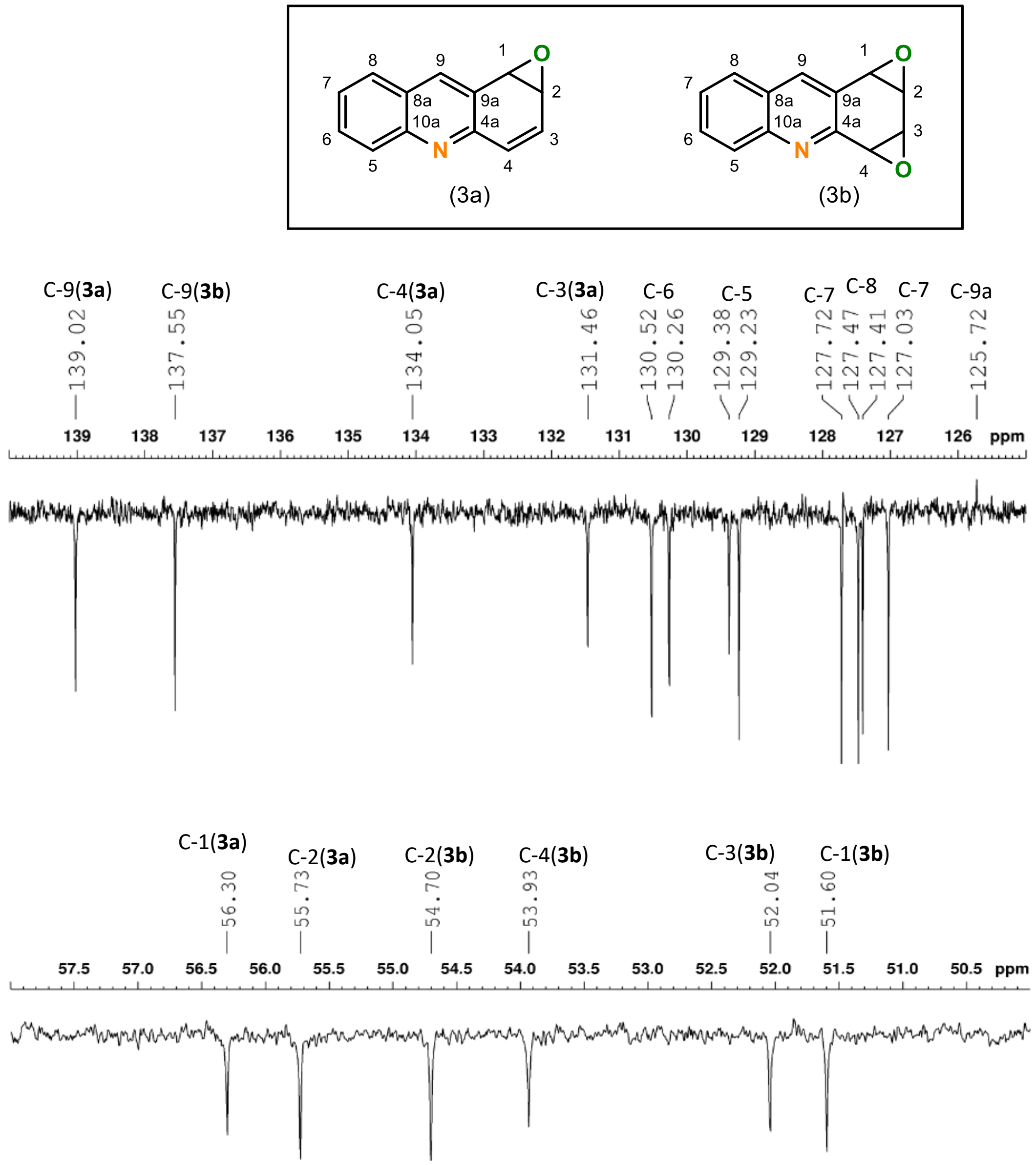

Figure S14. APT spectrum from a fraction containing a mixture of products **3a** and **3b** in $CDCl_3$.

5. Catalyst stability studies

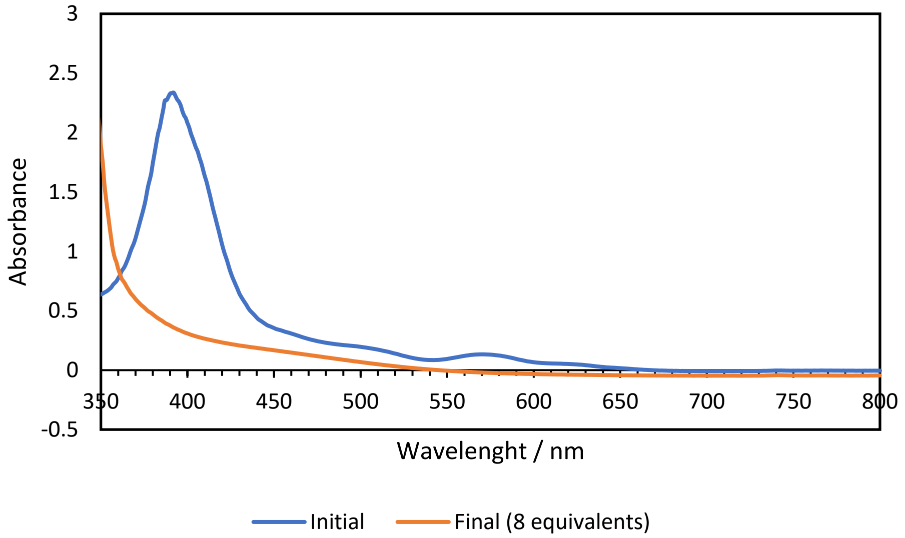

Figure S15. UV-vis of quinoline reaction mixture in $CHCl_3$. The bands observed in the initial spectrum are from the catalyst [Fe(TPFPP)Cl]. The Soret band is observed at ~400 nm.

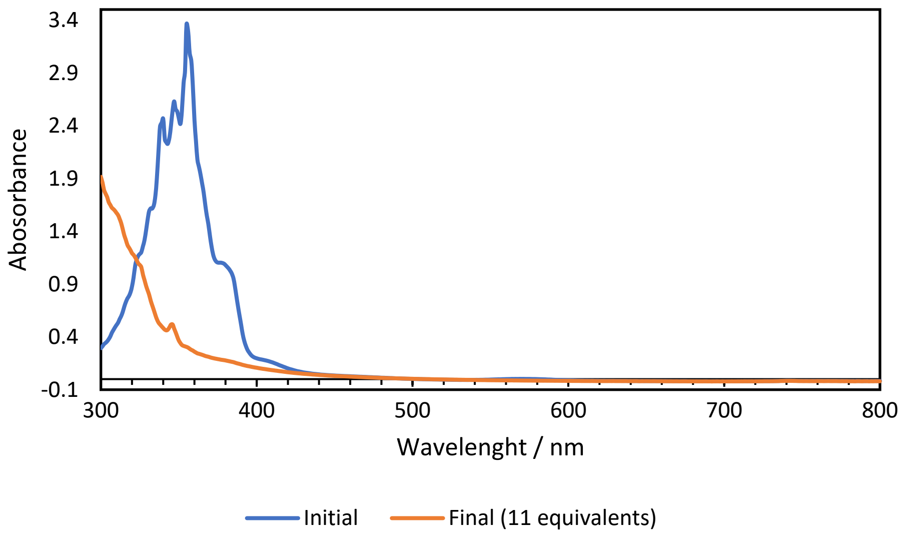

Figure S16. UV-vis of acridine reaction mixture in $CHCl_3$. The bands observed in the initial spectrum are from acridine (more intense bands) and the catalyst [Fe(TPFPP)Cl] (Soret band at 410 nm).
